# Plasma cell mastitis: a comprehensive review of etiological advances and future directions

**DOI:** 10.3389/fimmu.2026.1726988

**Published:** 2026-06-08

**Authors:** Hong Xu, Buxilite Da, Yuanqing Li, Hao Zheng, Lihua Xue, Fengxia Zhao, Yajing Wang, Lihua Hou, Xing Wang

**Affiliations:** 1 Department of General Surgery, The First Medical Center of the PLA General Hospital, Beijing, China; 2 Department of General Surgery, Jilin People’s Hospital, Jilin, China; 3 Department of General Surgery, The Third Medical Center of the PLA General Hospital, Beijing, China; 4 Beijing National Laboratory for Molecular Sciences, Institute of Chemistry, Chinese Academy of Sciences, Beijing, China

**Keywords:** diagnosis, etiology, immunity, mammary duct, plasma cell mastitis

## Abstract

Plasma cell mastitis (PCM), a complex and refractory chronic inflammatory-like breast disease, remains a critical global female healthcare challenge. Over the past decade, although considerable effects have been gained on understanding of the clinical and pathological features of PCM, the exact cause of PCM remains clinically challenging, as the pathogenesis of this disease involves multiple factors. This narrative review systematically synthesizes current evidence on PCM, mainly focusing on its etiology, clinical manifestations, diagnosis, and treatment strategies, which aims to unravel the etiological landscape of PCM from ductal microecology to systemic autoimmunity. We comprehensively demonstrated the etiology of PCM including the anatomical factors, immunological factors, microbial infections, and other factors. Anatomically, inverted nipples and duct structural abnormalities lead to a pathological chain of ductal obstruction - dilation - rupture - stromal inflammation; Immunologically, pro-inflammatory factors such as interleukin-6 (IL-6), tumor necrosis factor-α (TNF-α), interleukin-1β (IL-1β), and interleukin-17 (IL-17) are significantly elevated, and helper T cell 1 (Th1)/Th17 cells are overly activated, supporting the possibility that PCM may be an immune-mediated disease; The role of microbial factors is controversial, with most cultures being sterile and a few associated with anaerobic bacteria or mycobacteria; Smoking and obesity are involved in the pathogenesis through hormonal disorders and chronic inflammation. This review also provides diagnostic criteria and key points for differential diagnosis of PCM, and summarizes the current treatment strategies mainly based on glucocorticoids and surgery. Future research needs to establish reliable animal models and conduct multi-omics studies to reveal the complete causal chain.

## 1 Introduction

Plasma Cell Mastitis (PCM) is a complex and difficult-to-treat chronic inflammatory-like breast disease, currently classified as a refractory disease (1–3). In recent years, the incidence of PCM has increased, accounting for 2% of breast diseases (1). Once the disease occurs, it progresses rapidly and the lesion area is large. The current challenging issues regarding this type of disease are the difficult diagnosis, inconsistent naming, non-standardized and non-unified treatment methods, high recurrence rate, severe postoperative breast deformity, and controversial treatment approaches (4–6). Importantly, since the pathogenesis of this disease involves multiple factors, its exact cause has not yet been fully clarified. For example, anatomically, abnormal nipple and duct structure can lead to the onset, and secretion stasis causes ductal obstruction and dilation. In terms of immunology, it may be a self-limiting immune disease, related to autoimmune reactions, involving a variety of immune factors. Microbial infection factors are currently controversial, and some studies suggest that they may be related to anaerobic bacteria and mycobacteria. In addition, smoking, obesity, and other factors may also lead to hormonal disorders that damage the milk ducts and participate in the pathogenesis. Given the complex etiology of PCM and its expanding therapeutic applications, there is a pressing need to analyze recent developments and emerging trends in this field systematically. This review aims to combine the latest research progress in the etiology of plasma cell mastitis at home and abroad, providing comprehensive insights into the current state of the PCM, with particular emphasis on discovering the etiology of PCM from key aspects such as anatomical factors, immunological factors, microbial infection, and other factors, as well as their clinical implications. We also conclude the critical discussion of current challenges and prospects, highlighting the promising directions and scientific basis for future research and development that could lead to more effective diagnosis and therapeutic solutions in clinic.

## 2 Methodological description

This study is a narrative review rather than a systematic review. The literature search strategy is using keywords such as “plasma cell mastitis”, “periductal mastitis”, “etiology”, “immunology”, “treatment”, etc., to search PubMed, China National Knowledge Infrastructure (CNKI), and Wanfang Database. The time range is from the establishment of the database to December 2024. Inclusion criteria: clinical studies, basic research, and reviews related to the etiology, diagnosis, and treatment of PCM; exclusion criteria: non-English or non-Chinese literature, conference abstracts without full text available from the abstract alone, and case reports (unless they have significant implications). A total of 85 articles were included. This review comprehensively describes and qualitatively analyzes the evidence from the included literatures without conducting quantitative meta-analysis.

## 3 Diagnosis and differential diagnosis

### 3.1 Diagnostic criteria for PCM

**Figure 1:**
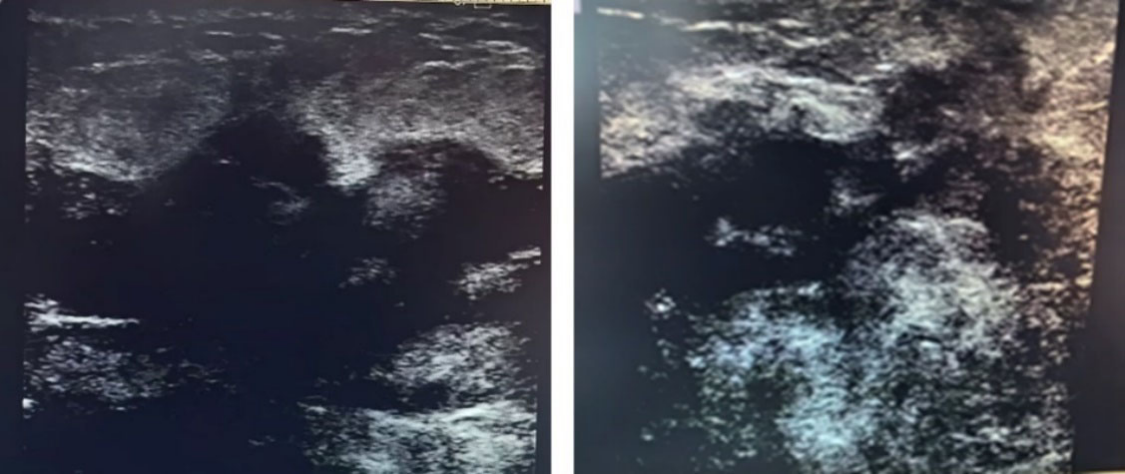
The pus cavity showed a “slippery sand-like” change.

**Figure 2:**
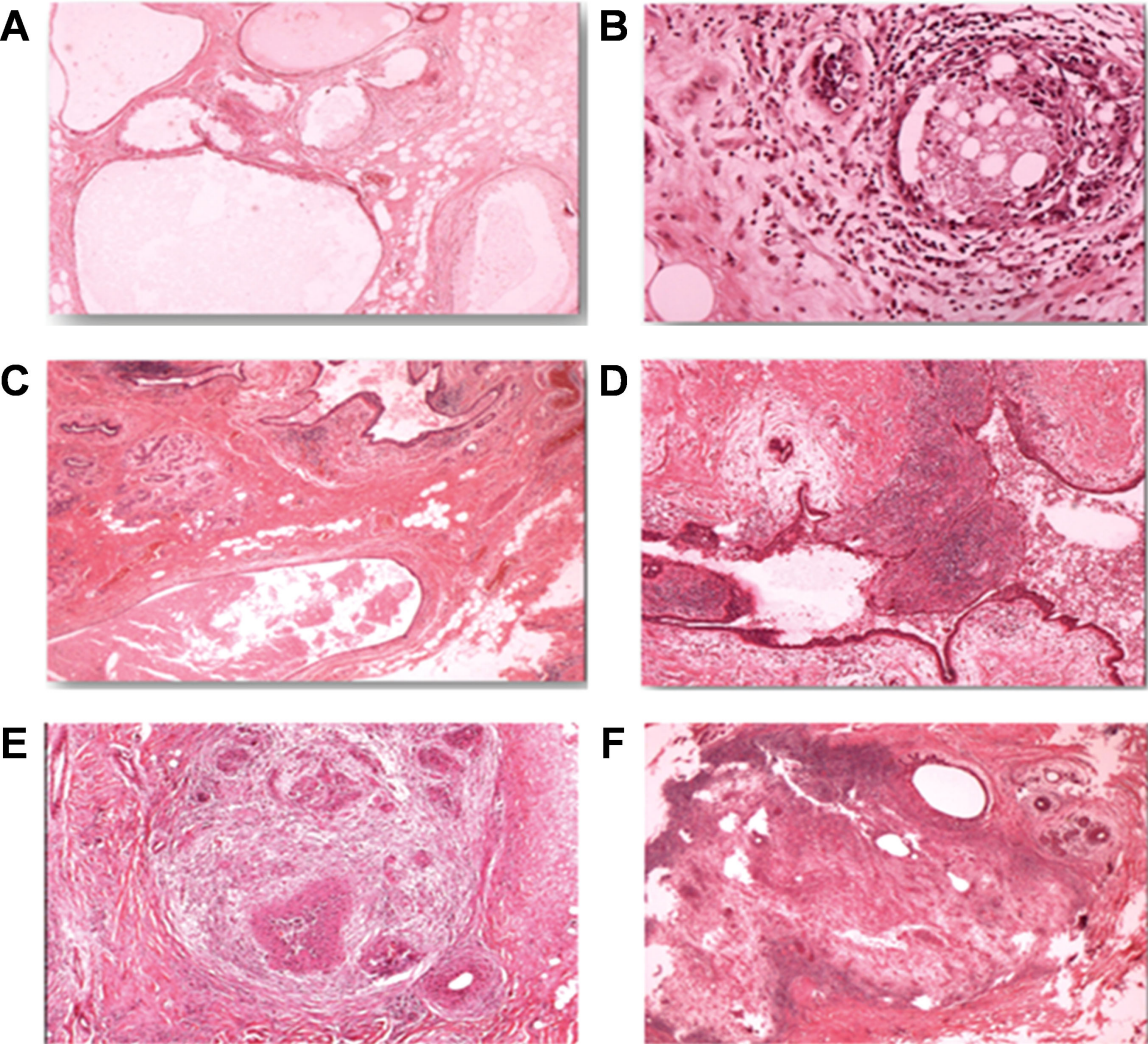
Diagram of histological staining for **(A)** Duct dilation; **(B)** Inflammatory cell aggregation around the dilated duct; **(C)** Duct dilation with inflammatory cell aggregation around the duct; **(D)** Inflammatory cells in the duct lumen and epithelial damage on the duct surface; **(E)** Partial damage to the breast ducts; and **(F)** Complete disintegration of the original ductal structure.

At present, there is no unified international diagnostic standard for PCM. Based on the literature and clinical practice, the following diagnostic points are recommended. 1) Clinical manifestations: Non-lactating women, often with a history of nipple inversion, presenting with breast masses, pain, nipple discharge (mostly serous or purulent), and recurrent episodes leading to abscesses or sinus tracts; 2) Imaging: Breast ultrasound shows duct dilation and thickened duct walls, accompanied by irregular hypoechoic areas; mammography (mammography) reveals blurred shadows around the ducts, without typical malignant calcifications. For example, **Figure 1** showed a typical ultrasound images of PCM of the breast, which displayed a “slippery sand-like” change in the abscess cavity; 3) Pathology: Core needle biopsy or surgical specimen histological examination shows breast duct dilation, dense infiltration of plasma cells (CD138+, CD38+) around the ducts, along with lymphocytes, neutrophils, and tissue cells, without granulomatous nodules or caseous necrosis. As shown in **Figure 2**, the histopathological examination confirmed the staged development of the ductal lesions. In the early stage, one or more main ducts were dilated, filled with thick secretions, and surrounded by a lymphocyte and plasma cell ring around the ducts (**Figures 2A–C**). The ductal epithelium was usually thin but still intact. In the middle stage, obvious epithelial damage and partial rupture of the duct wall occurred, resulting in the leakage of duct contents into the surrounding connective tissue (**Figures 2D, E**). The inflammatory infiltration became more diffuse and mixed, and occasionally formed granulomas. In the late stage, the original ductal structure was completely decomposed, replaced by diffuse granulomas and fibrotic reactions, containing a large number of plasma cells (**Figure 2F**). The remaining part of the ductal epithelium was difficult to identify. This pathological continuity provided a structural basis for the clinical staging of PCM.

### 3.2 Differential diagnosis

PCM needs to be clearly distinguished from the following diseases in **Table 1**. Of note, in clinical practice, it is recommended to perform hollow needle biopsy on all patients suspected of having PCM, rather than relying solely on imaging or clinical manifestations for diagnosis.

**Table 1:** Classification and characteristics of disease diagnoses with PCM.

Disease	Key differentiating characteristics
Idiopathic granulomatous mastitis	The pathology is non-caseous necrotic granuloma, mainly consisting of multinucleated giant cells and epithelioid tissue cells, with relatively few plasma cell infiltrations; it is usually not accompanied by nipple retraction (7, 8)
Tuberculous mastitis	Pathologically, there are caseous necrosis and acid-fast staining positive bacilli; clinically, there may be low fever and night sweats; the γ-interferon release assay (IGRA) is positive; anti-tuberculosis treatment is effective (9)
Breast cancer	The mass is hard in texture with unclear boundaries and poor mobility; imaging shows spiculation sign and clustered calcification; pathology reveals malignant cells (10, 11)
Acute bacterial mastitis	This is more common during lactation. The pathogenic bacteria are mostly Staphylococcus aureus. There is local redness, swelling and pain. Antimicrobial treatment is effective

## 4 Etiological factors

### 4.1 Anatomical factors

#### 4.1.1 Abnormalities in nipple and mammary duct structure

Clinical observations have showed that most patients with PCM have abnormal breast duct structures. Common symptoms include nipple depression, nipple shortening, and congenital developmental malformations, which can be categorized into several types, such as ductal inversion at the nipple site, ductal dilation and distortion, and ductal stenosis or interruption. These structural abnormalities impair the normal drainage of milk through the mammary ducts, leading to the accumulation of secretions. Inverted nipples often exhibit keratin plugs at the ductal openings, composed largely of keratin fragments (approximately 62% of the total mass) and bacterial biofilms (primarily consisting Staphylococcus epidermidis). These substances accumulate in a moist, obstructed environment and appear in a mucoid form, which then retrogradely enters the mammary ducts. This obstruction hinders the excretion of metabolic products, causing them to accumulate within the ducts, forming a “paste,” ultimately leading to the ductal blockage and the ductal dilation (12). Xing et al. also proposed that nipple inversion or deformities, ductal stenosis, and atypical mammary gland hyperplasia may lead to the nipple ductal obstruction, metabolic product excretion blockage, or ductal dilation (13). Xu et al. found that among 470 patients with PCM, 163 cases (34.7%) had nipple inversion, and those who did not have the condition corrected during the surgery had a significantly higher recurrence rate (14). In addition, this study also emphasized the high recurrence rate when surgical correction of nipple inversion was not performed. Conversely, Zhang et al. reported that traditional Chinese medicine treatment for nipple inversion achieved favorable results without the need for surgery, and recurrence rates were significantly reduced (15). Wang et al. further noted that nipple inversion occurs at a higher incidence in PCM patients (16).

The mammary duct, as the core structure of the breast, plays a critical role in the secretion and drainage of milk. Damage to the ductal structure not only directly disrupts the physical barrier of the mammary gland but also increases the permeability of the ducts. This leads to the displacement of retained substances from the ducts into the surrounding tissue. It is reported that the proposed mechanism of PCM development was that after ductal narrowing or obstruction, the ducts dilate, and the integrity of the ductal epithelium was compromised (17). As secretion within the duct increases, the pressure within the duct rises, causing the ductal walls to thin and undergo degenerative changes. In the early stages, ductal epithelial atrophy and ductal dilation occur. If the pressure within the duct continues to increase, the duct wall becomes further thinned, and a large amount of fluid in the duct will seep into the interstitial tissue, triggering an inflammatory response and even causing some necrotic lesions.

#### 4.1.2 Mammary duct obstruction

Mammary duct obstruction is commonly caused by excessive secretion of fluid within the ducts, with some patients manifesting as nipple discharge. Several studies suggest that elevated prolactin levels can result in the accumulation of substantial amounts of fluid in the ducts, which may lead to ductal obstruction and are often an early symptom of PCM onset. Some studies even propose that PCM induces temporary hyperprolactinemia, typically lasting for about 3 weeks after disease onset (18). In a study involving 700 PCM patients, 380 cases presented with nipple discharge, accounting for half of the total cases. As shown in **Table 2**, these cases were classified into three categories based on common causes: the first category being patients with microadenomas or elevated prolactin levels, the second category involving patients who had elevated prolactin due to the use of antidepressants, antipsychotic drugs, antihypertensives, or gastrointestinal medications, and the third category consisting of patients with residual milk after pregnancy termination or inadequate weaning, where 70% of the cases belonged to the third category (19).

**Table 2:** The classification of nipple discharge.

Type	Cause	Proportion
Pituitary tumor or idiopathic hyperprolactinemia	Microadenoma of the pituitary gland or other non-specific causes	10%
Drug-induced increase in prolactin	Intake of oral antidepressants, psychotropic drugs, hypertension medications or stomach medications	15%
Pregnancy/lactation	Termination of pregnancy or cessation of breastfeeding without weaning and/or possible residual breast milk	75%

The composition of breast milk also plays important roles in the pathogenesis of mastitis. Milk components such as fats, proteins, and lactose significantly influence the physiological function of the mammary glands and the secretion. The prolonged retention of residual milk in the mammary ducts increases the viscosity over time, leading to physicochemical changes to form the “greasy” substances. These substances are highly viscous and easily adhere to the duct walls, contributing to narrowing or even complete obstruction of the ductal lumen. Additionally, excessive milk retention leads to a continuous increase in the amount of fluid overflowing in the milk ducts, increasing the pressure within the ducts. The inner walls of the milk ducts gradually undergo atrophy and thinning, and the ductal epithelium shrinks. Subsequently, there will be a situation of ductal dilation. As the internal pressure of the duct continues to increase, the duct wall will become even more fragile, eventually leading to rupture. A large amount of fluid will seep into the interstitial space, thereby triggering an inflammatory response (20). Notably, the changes following ductal dilation mirror those observed in the earlier mechanism of nipple inversion.

### 4.2 Immunological factors

#### 4.2.1 Immune factors and mediators

To enhance reproducibility, the data summarized in this section are all derived from the results obtained by using immunohistochemistry (IHC), enzyme-linked immunosorbent assay (ELISA), real-time fluorescence quantitative PCR (RT-PCR), etc. to test the tissues or serum samples of PCM patients, and are compared with normal breast tissues or healthy controls. Each study has a control group, and the specific sample size and statistical processing can be found in the original literature. The data standardization process refers to the reference internal control genes (e.g., GAPDH, β-actin) or protein quantification methods used in each literature. A single-cell sequencing study thoroughly examined the cells in normal breast tissue (21). The results indicated that the normal mammary duct microenvironment consists of pericytes, endothelial cells, monocytes/macrophages, T and B lymphocytes, and fibroblasts. These immune cells contribute to chronic inflammation and immune surveillance. They are also capable of recognizing new antigens, as well as endogenous and exogenous ligands. A characteristic feature of PCM is the extensive infiltration of plasma cells. Plasma cells originate from B cells, which are derived from hematopoietic stem cells in the bone marrow and express surface molecule CD20 (22). The differentiation and function of plasma cells are influenced by various cytokines, among which IL-6 is particularly important (23, 24).

Additionally, epithelial-mesenchymal transition (EMT) is reported as a central driver of PCM pathogenesis, intricately regulated by disease-specific immune microenvironment. The autoimmune-mediated DE initiates the ductal damage, generating damage-associated molecular patterns (DAMPs) that activate pattern recognition receptors (PRRs). This triggers NF-κB signaling hubs, upregulating pro-inflammatory mediators (IL-1β, IL-6, TGF-β1, ICAM-1, CXCL12) and core EMT-transcription factors (Snail, TWIST). Crucially, IL-6/JAK/STAT3 signaling promotes plasma cell survival via Bcl-2 and drives EMT in ductal epithelium. Concurrently, IL-1β activate PI3K/Akt to stabilize EMT effectors and enhance ECM synthesis (25). Based on current research of serum and tissue immunohistochemistry analyses, there are numerous reports on the occurrence of PCM and its related immune factors. They can be categorized into five main groups based on their functional roles, including pro-inflammatory/anti-inflammatory cytokines, Th subgroup-related factors, chemokines, immune cell markers, and autoantibodies. This classification covers core mechanisms of PCM immune imbalance (e.g., inflammation, immune regulation, cellular infiltration, autoimmunity) and their association with the pathological mechanisms of PCM (e.g., plasma cell infiltration, ductal dilation, and fibrosis) or clinical features (e.g., inflammatory activity and symptom severity). The synergistic effects of PCM with various immune factors are summarized in **Tables 3**, **4**. Wherein, “significant increase” refers to a statistically significant difference when compared with the normal control group. The “—” indicates that there is currently no clear quantitative data or research reports.

**Table 3:** The synergistic effects of PCM and inflammatory factors.

Categories of Immune Factors		The main role in PCM	Expression changes (vs normal breast)	Clinical significance or correlation
Anti-inflammatory cytokines	IL-10	Inhibit the release of pro-inflammatory cytokines (such as TNF-α, IL-1β), regulate immune response, and limit the excessive spread of inflammation	Slight increase or no change (P > 0.05)	It may not be able to compensate adequately
	TGF-β	Inhibit lymphocyte proliferation and promotes tissue repair, however, excessive expression can induce fibrosis, resulting in stiffening and occlusion of the mammary ducts	Increase (P < 0.05)	It is related to the stiffness and occlusion of the catheter
	IFN-β	Inhibit the activation of macrophages, reduce the release of pro-inflammatory factors such as TNF-α and IL-1β, block the activity of the NF-κB pathway	Not clear	—
Pro-inflammatory cytokines	TNF-α	Activate the endothelial cells, promoting the infiltration of inflammatory cells, induce damage to mammary ductal epithelial cells, aggravating the ductal dilation and inflammatory responses	Significant increase (P < 0.01)	It is positively correlated with the severity of PCM inflammation and can be used as an indicator of disease activity and the potential therapeutic targets (such as anti-TNF-α agents)
	IL-1β	Activate the immune cells (such as macrophages and lymphocytes), promote the release of inflammatory mediators like prostaglandins, and intensify local redness, swelling, heat and pain	Significant increase (P < 0.01)	It participates in the acute inflammatory response of PCM, and the higher-level leads to more obvious pain and lump symptoms
	IL-6	Promote the differentiation of B cells into plasma cells (resulting in the characteristic plasma cell infiltration of PCM), induce the synthesis of acute-phase proteins and maintain the chronic inflammatory state	Significant increase (P < 0.01)	It is related to the chronic progression of PCM. The level of serum IL-6 can assist in evaluating the activity of disease.
	IL-8	Chemoattract neutrophils and monocytes to the inflammatory site, promoting tissue infiltration and destruction, participating in the formation of inflammation around the mammary ducts	Significant increase (P < 0.01)	It reflects the degree of local inflammatory cell infiltration and is related to the size and texture of the PCM mass.
	IL-2	Promote the expansion of T cells (especially CD8+ T cells and Tregs), with a priority on the expansion of Tregs	Not clear	—

**Table 4:** The synergistic effects of PCM and other related factors.

Categories of Immune Factors		The main role in PCM
Th1/Th2-related cytokines	IFN-γ	The representative factor of Th1 cell activates macrophages, enhances cellular immunity, and participates in the immune damage of ductal epithelial cells
	IL-4	The representative factor of Th2 cells promotes B cell activation and antibody production, and inhibits excessive activation of Th1 cells
	IL-13	The representative factor of Th2 cells promotes the proliferation of fibroblasts and collagen synthesis, and is involved in the fibrosis of breast tissue
Th17-related cytokines	IL-17A	The representative factor of Th17 cells, strongly chemoattracts neutrophils, promotes the release of pro-inflammatory factors (IL-6, IL-8), and exacerbates tissue inflammation
	IL-23	Promote the differentiation and maintenance of Th17 cells, and amplify the inflammatory response mediated by IL-17A
Chemokines	CXCL8 (IL-8)	Chemoattract neutrophils
	CCL2 (MCP-1)	Chemoattract monocytes and T cells to the inflammatory site, promote the activation of macrophages, and participate in the formation of chronic inflammation.
	CXCL10 (IP-10)	Induced by IFN-γ, the chemotactically activated T cells (Th1 cells) amplify the inflammatory damage mediated by cellular immunity.
Immune cell surface markers	CD4+ T cells/CD8+ T cells	CD4+ T cells (helper T cells) dominate the immune response, while CD8+ T cells (cytotoxic T cells) are involved in cell killing.
	CD19+ B cells/CD138+ plasma cells	CD19+ B cells differentiate into CD138+ plasma cells, which secrete antibodies (such as immunoglobulins) and form immune complex deposits.
	Foxp3+ T regulatory cells	Regulatory T cells, inhibit excessive immune responses and maintain immune tolerance
Autoantibody	Antinuclear Antibody (ANA)	Autoantibodies targeting the components of the cell nucleus suggest the involvement of an autoimmune response
	Anti-breast ductal epithelial antibody	Specific autoantibodies against breast ductal epithelial cells may mediate epithelial cell damage

According to current literatures on PCM and immune mediators, the most extensively studied cytokines include interleukin-6 (IL-6), interleukin-17 (IL-17), tumor necrosis factor-α (TNF-α), and interleukin-1β (IL-1β), which together account for approximately 75%. Among these, IL-6 is a pro-inflammatory cytokine and a pivotal driver of Th17 cell differentiation. It stimulates the production of acute-phase proteins, including C-reactive protein (CRP), thereby playing a central role in immune and inflammatory processes (26). Liu et al. demonstrated that IL-6 promotes the differentiation of B cells into plasma cells and antibody secretion via activation of the IL-6/JAK2/STAT3 signaling pathway, which can induce PCM in murine models (27). When IL-6 binds to its receptor (IL-6R) on the cell surface, Janus kinase (JAK) is phosphorylated, leading to phosphorylation of STAT3. This activation subsequently promotes the expression of the anti-apoptotic protein Bcl-2 while inhibiting plasma cell apoptosis. On the other hand, the activated JAK can also trigger the PI3K/Akt/mTOR pathway to induces the inflammatory responses, which plays an important role in the pathogenesis of PCM (21).

IL-17, predominantly secreted by the Th17 cell subset, represents another key pro-inflammatory mediator, with IL-17A and IL-17F being the most extensively studied isoforms. IL-17 acts as an early initiator of T-cell-mediated inflammatory responses, inducing neutrophil recruitment. It also stimulates endothelial cells, epithelial cells, and fibroblasts to secrete IL-6, IL-8, granulocyte colony-stimulating factor (G-CSF), and prostaglandin E2 (PGE2), while simultaneously enhancing the effects of IL-1 and TNF-α. These actions collectively amplify inflammatory cascades and contribute to tissue injury (28, 29). So, IL-17 plays a critical role in the initiation and progression of inflammation.

#### 4.2.2 Self-limiting immune-mediated disease

With advances in molecular immunology and molecular biology, notable progress has been achieved in the diagnosis, therapeutic strategies, and efficacy assessment of PCM. Some researchers have proposed that PCM may represent a self-limiting immune-mediated disease. In particular, the Th17 cells have been implicated in several autoimmune disorders, and their hyperactivation has been linked to rheumatoid arthritis and psoriasis (30–34). However, to date, no definitive objective evidence has established a direct causal link between PCM and autoimmune disease mechanisms.

Previous studies have long recognized T helper (Th) cells as the principal effector cells in a wide range of autoimmune diseases (35). Among these, Th1 cells are distinguished by their production of interferon-γ (IFN-γ), which enhances cellular defense against intracellular infections. Liu et al. reported that both IFN-γ and interleukin-12A (IL-12A) are significantly upregulated in PCM breast tissue (36). Importantly, IFN-γ and IL-12A act synergistically to activate the Th1 subset, suggesting that excessive Th1 activation may be present in PCM patients.

### 4.3 Microbial infection factors

A large number of retrospective bacteriological studies suggest that bacterial cultures from PCM lesions are generally negative, supporting the view that PCM is essentially a sterile inflammatory condition (37–39). In our previous study, pus aspirates from 87 cases were cultured, with only one case yielding *Mycobacterium-like* organisms and one case yielding anaerobes, while no other bacterial pathogens were identified (19). These findings indicate that although PCM itself usually does not cause infection, it cannot completely rule out the possibility of secondary infection.

Nevertheless, more recent studies have reported positive culture results from PCM tissues or pus, including *Staphylococcus aureus* (40). Xing et al. reported that when PCM progresses to abscess formation, a variety of microorganisms such as *Enterococcus* spp., *anaerobic streptococci*, *Staphylococcus aureus*, *Bacteroides*, and *nontuberculous mycobacteria* can be isolated (13). These pathogens may promote the release of inflammatory cytokines and chemokines, trigger innate immune responses, recruit lymphocytes, and ultimately lead to tissue necrosis and abscess formation. Historically, some scholars have proposed *Mycobacterium tuberculosis* infection as an etiological factor, and empirical anti-tuberculosis treatment has been administered (41–43). To date, certain medical institutions still apply anti-tuberculosis therapy in PCM patients, however, the therapeutic efficacy remains uncertain. Large-scale cohort studies are still lacking and urgently needed.

### 4.4 Smoking, obesity, and other risk factors

Cigarette smoking exerts broad and complex effects on breast tissue. It has been identified as a significant risk factor for PCM (44). Smoking can damage the structural and functional integrity of mammary ductal epithelial cells. Dixon and Bundred reported that tobacco degradation products, including lipid peroxides and nicotine derivatives, may cause estrogen-progesterone imbalance, ductal injury, and obstruction, which are important risk factors for PCM recurrence and disease progression (45, 46). These hormonal changes can further predispose breast tissue to damage, favor anaerobic bacterial growth, and amplify local inflammatory responses, thereby culminating in suppurative infection. Epidemiological studies also supported this association, prospective cohort studies showed that smokers had a significantly higher risk of developing mastitis compared with non-smokers. Both *in vivo* and *in vitro* studies demonstrated that smoking could stimulate the release of inflammatory mediators and enhance the cytokine expression, thereby promoting ductal obstruction and plasma cell infiltration.

Obesity has also been proposed as a risk factor for PCM. Clinical investigations revealed that most patients diagnosed with plasma cell mastitis were overweight or obese (47–49). Until now, Obesity is considered a chronic low-grade inflammatory state, which may alter the immune response and accelerate disease progression. However, the mechanistic link between obesity and PCM remains poorly defined and requires further investigation.

## 5 Current treatment landscape and recurrence rate

Although the present study primarily focuses on etiological factors, a brief overview of current therapeutic strategies (e.g., conservative treatment and surgical resection) for PCM is simply summarized in response to the clinic need. On the one hand, the conservative management includes several methods (8, 50). 1) Systemic corticosteroids, such as oral prednisone or methylprednisolone (starting dose 20 mg/day with gradual tapering), are commonly used to suppress inflammation and reduce mass size; however, a high relapse rate is frequently observed upon drug discontinuation. 2) Non-steroidal anti-inflammatory drugs (NSAIDs) are also employed for pain relief. 3) Traditional Chinese medicine, including formulations like Yanghe decoction, has been investigated in some studies and may exert beneficial effects by modulating the local microecology (41). 4) Bromocriptine, a dopamine agonist, is specifically indicated for patients with hyperprolactinemia and has been shown to lower the risk of recurrence (21). On the other hand, surgical resection remains the mainstay of treatment for patients who are refractory to medical therapy, experience recurrent episodes, or develop abscesses or fistulous tracts. A critical determinant of surgical success is the simultaneous correction of pre-existing nipple retraction. Evidence indicates that failing to correct nipple inversion during surgery is associated with a significantly higher postoperative recurrence rate (14). It was noted that the recurrence rates for PCM generally range from 15% to 30%, and recurrence is more frequent after conservative treatment alone compared with surgical resection combined with concurrent nipple correction. This disparity underscores the importance of addressing both the inflammatory process and underlying anatomical predisposition to achieve durable disease control.

## 6 Conclusion and future perspectives

This review systematically synthesizes current evidence on the etiology of PCM. It is increasingly clear that PCM pathogenesis is multifactorial due to the interplay of anatomical abnormalities, immune dysregulation, microbial factors, and lifestyle-related influences. Within this complex landscape, the present review makes several key contributions. First, we delineate a progressive anatomical-pathological chain of ductal obstruction - dilation - rupture - stromal inflammation, which provides a unified mechanistic framework for understanding disease initiation and progression. Then, we summarize the expression profiles of major immune factors implicated in PCM (including IL-6, IL-1β, TNF-α, and others) and the laboratory methods used for their detection (immunohistochemistry, ELISA, RT-PCR). These findings lend supportive evidence to the hypothesis that PCM is, at least in part, an immune-mediated disorder. Furthermore, we have also integrated practical diagnostic and differential diagnostic criteria, aiming to reduce clinical misdiagnosis situations - given that PCM exhibits characteristics similar to those of breast carcinoma. Finally, we highlight a straightforward but clinically impactful recommendation: concurrent correction of nipple retraction during surgical treatment, as failure to do so is associated with significantly higher recurrence rates.

Despite these insights, current diagnostic and therapeutic practices for PCM remain highly heterogeneous, and recurrence rates are unacceptably high. A thorough understanding of the pathogenesis of PCM is of great significance for clarifying its causes and guiding clinical treatment (51–57). Therefore, several important gaps need to be addressed before the proposed framework can be fully translated into clinical practice. The future direction of PCM etiology research will undoubtedly shift from macroscopic clinical observation to the microscopic molecular and cellular world, and ultimately construct a multi-factor, dynamic interaction-based systemic etiology model. The core will revolve around the main axis of “genetic susceptibility - duct microenvironment imbalance - abnormal immune response”, and leverage cutting-edge technologies for in-depth and extensive exploration.

First, a major limitation of the field is the lack of a reliable animal model that recapitulates the full histopathological spectrum of human PCM. Without such a model, *in vivo* testing of causal mechanisms, for example, whether isolated ductal rupture is sufficient to initiate the disease, remains impossible. Future efforts should therefore focus on developing rodent models that reproduce the progressive ductal dilation, rupture, and stromal inflammation seen in patients. Such models would enable controlled intervention studies and provide a platform for testing novel therapeutics. Second, the molecular networks driving PCM are still poorly defined. Multi-omics approaches including single-cell transcriptomics, metabolomics, and microbiome profiling applied to well-staged patient tissues could systematically delineate the cellular heterogeneity and signaling pathways involved. However, these techniques have not yet been systematically applied to PCM. If successful, they might identify novel diagnostic biomarkers (e.g., specific cytokine signatures) or therapeutic targets (e.g., key nodes in the IL-6/JAK/STAT3 axis). Third, although immune dysregulation has been implicated, the precise mechanisms such as which antigens trigger the response, how autoantibodies develop, and whether the process is T-cell driven remain unknown. Functional cellular and molecular experiments are needed to validate the exact contribution of candidate pathways. Addressing these questions can open the door to targeted immunomodulatory therapies that are more effective and less toxic than broad-spectrum corticosteroids. Fourth, the clinical evidence base for PCM treatment is weak. Prospective, well-controlled trials are urgently required to evaluate the long-term efficacy and recurrence rates of existing modalities (corticosteroids, bromocriptine, surgery, and biologics) and to establish an individualized etiology-based stratification strategy. For example, it remains unknown whether patients with hyperprolactinemia benefit more from bromocriptine than from steroids, or whether surgical correction of nipple retraction yields durable recurrence reduction across all disease stages. In addition, development of multimodal imaging-based diagnostic criteria and exploration of the interaction between environmental triggers and intrinsic factors will also be a focus to improve the accuracy of PCM pathogenesis. Fifth, emerging computational tools such as machine learning and artificial intelligence have shown promise in other areas of breast imaging and risk prediction (58–60). However, their application to PCM is still preliminary. Future research could explore whether AI algorithms, trained on high-quality, multicenter clinical and imaging data, can assist in differentiating PCM from malignancy or in predicting individual recurrence risk. At present, such applications remain open research perspectives rather than imminent clinical tools, and they should be pursued only after basic mechanistic and clinical questions have been better resolved. Collectively, addressing these research priorities from animal models and multi-omics to clinical trials and computational tools will be essential to transform the ductal injury-stromal response hypothesis into actionable improvements in patient care. Importantly, each of these directions requires hypothesis-driven, rigorous investigation rather than speculative forecasting.

Consequently, the exploration of fundamental causes of diseases will be the cornerstone for achieving breakthrough progress. By leveraging genomic, microbiomic, and proteomic technologies to conduct in-depth research on the complex relationships among autoimmune responses, hypersensitivity reactions triggered by intraluminal lipid secretions in milk ducts, and local microbial ecological imbalances, it is expected to reveal the pathogenesis of PCM and thereby identify key molecular targets for prevention and targeted treatment. Additionally, future studies should also focus on the roles of inflammatory mediators, cytokines, tissue repair, and fibrosis in PCM, which may help to unravel its complex pathophysiology.
